# The effectiveness of mandatory *v*. voluntary food reformulation policies: a rapid review

**DOI:** 10.1017/S0007114524001326

**Published:** 2025-03-28

**Authors:** Mathilde Gressier, Gary Frost, Zoe Hill, Danying Li, Jack Olney, Elisa Pineda, Victoria Targett, Michelle Young, Franco Sassi

**Affiliations:** 1 Section for Nutrition Research, Department of Metabolism, Digestion and Reproduction, Faculty of Medicine, Imperial College London, London, UK; 2 Centre for Health Economics & Policy Innovation, Department of Economics & Public Policy, Imperial College Business School, Imperial College London, London, UK; 3 Office for Health Improvement & Disparities, Department of Health and Social Care, London, UK; 4 The George Institute for Global Health UK, School of Public Health, Imperial College London, London, UK

**Keywords:** Food policies, Food reformulation, Evidence review

## Abstract

While reformulation policies are commonly used to incentivise manufacturers to improve the nutrient profile of the foods and beverages they produce, only a few countries have implemented mandatory reformulation policies. This paper aimed to review evidence on the design, implementation challenges and effectiveness of mandatory reformulation policies and compare them to voluntary reformulation policies. The systematic search retrieved seventy-one studies including twelve on mandatory reformulation policies. Most mandatory reformulation policies were aimed at reducing *trans*-fatty acids or sodium in foods. Overall, mandatory reformulation policies were found to be more effective than voluntary ones in improving dietary intakes. Mandatory policies were implemented when voluntary policies either failed or were found to be insufficient to improve the composition of foods. Typical features of mandatory policies could also improve the design of voluntary policies. Examples include strict but attainable targets and a tight monitoring of compliance.

Unhealthy diets are now a significant risk factor for poor health and premature death in the UK. Obesity and the resulting adverse health impacts cost the NHS annually about £7 billion^(1)^ (for comparison, high blood pressure costs annually about £2·1 billion^(2)^). One solution to help decrease the burden of unhealthy diets that has been implemented in the UK is a voluntary, industry-focused product reformulation programme, to prompt businesses to modify the composition of a food or drink to improve its nutrient content. Reformulation enables consumers to eat more healthily without them consciously having to change the everyday foods they buy and can give businesses a competitive advantage. Three workstreams have been implemented to promote and focus reformulation in key areas: salt reduction in 2006, sugar reformulation in 2016 and energy reduction in 2018. While regular product review and reformulation is undertaken by businesses in all sectors – retailers, manufacturers and eating out of home – as a matter of course, action to improve the nutrient composition of products through these regular cycles can be triggered by different policies. This can be directly with reformulation policies, as in the UK, or indirectly by, for example, fiscal policies or marketing restrictions. For example, the UK Soft Drinks Industry Levy was structured in such a way to encourage businesses to reformulate products and has resulted in average reductions in sugar levels per 100 ml of 46 % between 2015 and 2020, making products liable for a lower rate of tax or no tax at all. Setting mandatory standards for the composition of food and drink is a stringent way to control or improve their nutrient composition. When these are applied, manufacturers, retailers and eating out of home businesses must reformulate any products that do not meet the standards to ensure compliance or delist them. Examples include South Africa’s or Argentina’s mandatory salt legislation^(3,4)^; or, for example, the ban on *trans*-fatty acids (TFA) implemented in many countries has pushed manufacturers to change the type of oil used in bakery products, spreads and fried foods^(3–5)^. Compliance with mandatory reformulation programmes can be enforced where necessary, for example, through the use of fines. Whether a programme is mandatory or voluntary, these often involve category-specific standards for the salt, sugar or energy content of foods, such as those used in the UK, South Africa and Argentina^(6–8)^.

A published systematic review on the impact of worldwide reformulation policies (both mandatory and voluntary) on consumer behaviour, dietary intakes and health status found only five studies on mandatory reformulation policies, compared with fifty-four studies on voluntary reformulation policies^(9)^. All studies on mandatory standards studied the effect of TFA bans. The body of evidence showed that bans on TFA were associated with a reduction in mortality. Recently, new mandatory reformulation policies have been enforced on salt and TFA in various countries, and new evidence has been published. In addition, the previous published review did not include the effect that reformulation has on the nutrient profile of foods.

The aim of this evidence review was to gauge the effectiveness of mandatory reformulation policies specifically on businesses’ responses, on the quality of foods and any changes made to products, levels of compliance and people’s health, building on the findings of a previous systematic review^(9)^. The focus was placed on mandatory policies as the UK has had a voluntary programme in place since 2006 and much is already known about how these initiatives work, their impact, etc. A further goal was to compare the overall delivery from, and strengths and limitations of, mandatory policies to those of voluntary reformulation policies, and to discuss aspects of the design and implementation of mandatory reformulation programmes.

## Methods

This review uses, extends and updates evidence from a systematic review undertaken previously by some of the same authors involved in this paper. The search strategy for the original review included articles up to and including December 2018 and assessed evidence from evaluations of both voluntary and mandatory reformulation policies on consumer-related outcomes (behaviour, nutrient intake and health status)^(9)^. The current review extends the scope to include the effect of voluntary and mandatory reformulation programmes on food composition and includes papers up to April 2021.

### Search strategy

The search strategy was designed to retrieve papers with keywords corresponding to the concept of reformulation and an outcome corresponding to changes in food nutrient composition (e.g. sugar content in foods) or dietary intakes (e.g. average sugar intakes) or health status (e.g. prevalence of diabetes). Compared with the previous review, studies with the nutrient composition of foods as outcomes were identified from the search strategy and added to this review.

Studies were searched for using three databases: EMBASE, MEDLINE and Global Health, which are the same databases used in the original review. Papers dated up to April 2021 were searched for. The full search strategy run on MEDLINE, as an example, is in the online Supplementary material Table 1.

Reports and other elements of grey literature were searched for on Google, using a combination of selected keywords from the full search strategy used in the three databases and additionally using Boolean terms combining these terms. The reference lists of studies included in the previous review were also searched for additional papers to screen.

The NOURISHING database that gathers information on policies to improve diet and physical activity was used to build an overview of countries that have implemented reformulation policies, but not necessarily evaluated them^(10)^. All policies under the topic ‘reformulation’ were searched.

### Inclusion and exclusion criteria

The inclusion criteria for this review used the PICO (Population, Intervention, Comparison, Outcomes) framework and was adapted from the search strategy of the systematic review by Gressier *et al.* (2021)^(9)^ (Table 1). Reformulation policies that targeted foods for the general population were included (i.e. foods sold in retail or restaurants/other businesses in the eating out sector). Policies on branded foods sold in schools were also included, as these can be reformulated by their manufacturers, similarly to foods sold in retail.

**Table 1.** PICO (population, intervention, comparator, outcome) table for the selection of studies for the evidence review (adapted from Gressier *et al.*, 2021)^(9)^

PICO feature	Criteria
Population	General population, no geographical restriction. *Studies focusing on the effect in specific subgroups with medical conditions were excluded.*
Intervention	Reformulation interventions include those targeting packaged foods and/or non-alcoholic beverages, or food sold in restaurant chains/other businesses in the out-of-home sector. Fortification interventions were not included. Reformulation can be driven by policies designed with a border scope, such as taxation, labelling and others. Although in some instances such policies have been designed with the explicit goal of incentivising reformulation, among others (e.g. the Soft Drinks Industry Levy in the UK) these have not been included in this review because the goals of these policies are not always stated explicitly, and in most cases reformulation is not a primary goal.
Comparison	Comparators may include no intervention or a comparison of the same group before the implementation of the intervention.
Outcomes	Studies must focus on food composition, food product choices, nutrient intakes or long-term health outcomes linked to non-communicable diseases. Primary outcomes include choice behaviour (purchases and sales), dietary intakes and patterns, risk factors for non-communicable diseases (BMI, blood pressure and biological markers of dietary intakes) or health outcomes (mortality).

Selected studies evaluated the impact of reformulation policies on the composition of foods, consumer behaviour, dietary intake or health status of consumers. Studies that discussed the design or implementation of mandatory reformulation policies were also included. The focus of the reformulation policy could be any food component or nutrient, but fortification was not included in the definition of reformulation in this study. Data related to these elements were extracted and written as a narrative review. The narrative review focused on mandatory reformulation policies.

Studies published in English and in full text only were considered due to time limitations in conducting the review.

### Risk of bias assessment

A risk of bias assessment was performed in the earlier review, and this was used also in this review for the studies derived from the earlier one^(9)^. Based on the criteria used in the earlier review, the risk of bias profile for the additional studies included in this review was found to be in line with that of the earlier studies. Therefore, no new systematic assessment was carried out for the current study.

## Results

**Fig. 1.**
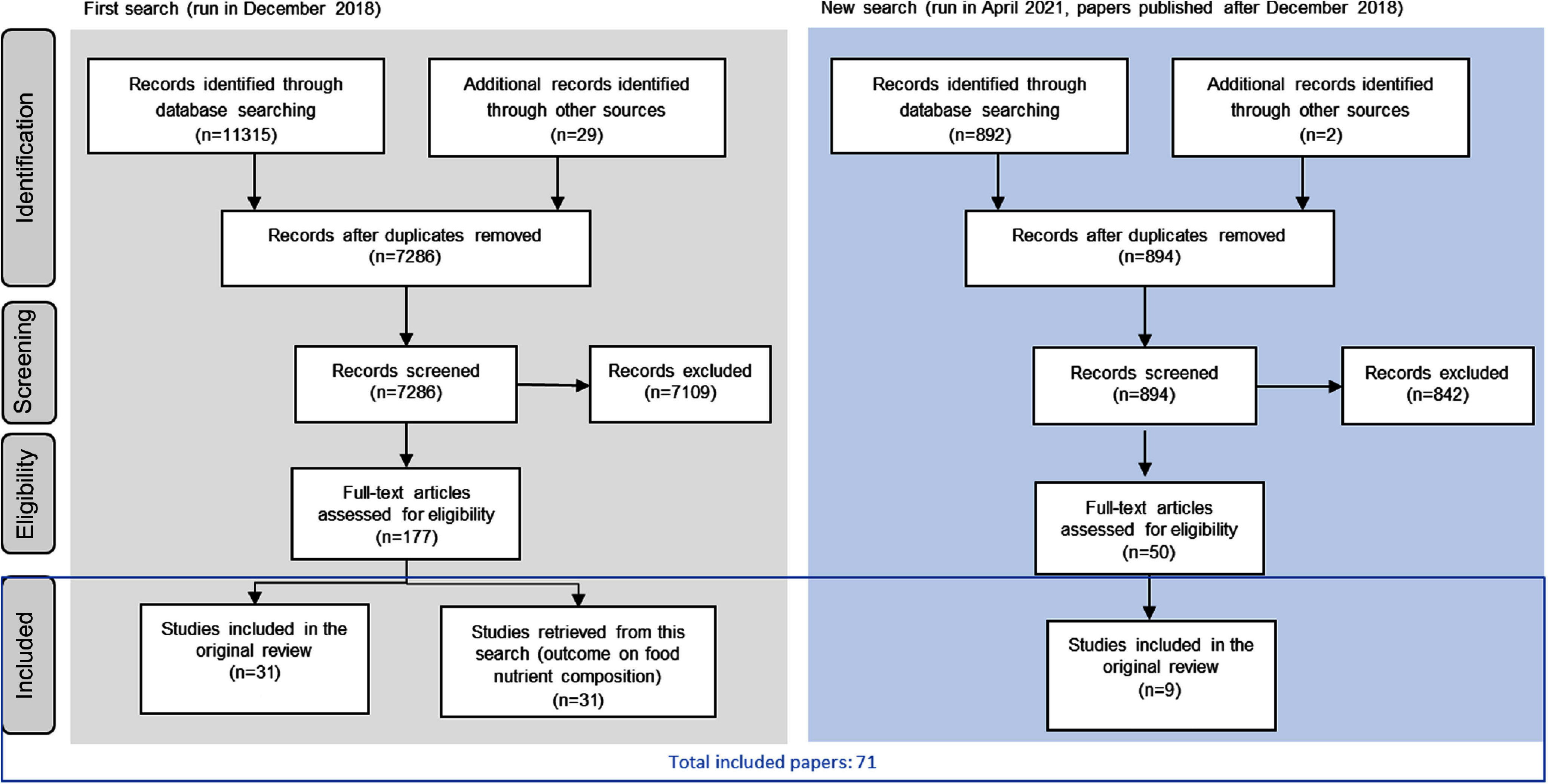
Flow chart showing the screening of records from the two search strategies (adapted from PRISMA^(29)^).

The search strategy retrieved seventy-one studies that were included in this review (Fig. 1). Twelve studies evaluated mandatory reformulation policies, while fifty-nine evaluated voluntary reformulation policies. All studies except one were published after 2000, and most were published after 2010. Compared with the previous systematic review, forty studies were added (thirty-one from the first search strategy and nine from the updated search).

### Risk of bias

The risk of bias assessment was available for the thirty-one studies included in the original review (43 % of those included in this review). There were few studies with a low risk of bias (online Supplementary Table 2). ‘Adjustment’ was a domain in which many studies showed a high risk of bias: their design did not allow for an assessment of the effect of reformulation isolated from the effect of other policies that may have been implemented concurrently. This means that the effects evaluated in this review may reflect aspects of the context in which reformulation is implemented, rather than reformulation alone.

### Effectiveness of mandatory reformulation policies

#### How common are mandatory reformulation policies?

Mandatory reformulation policies were found in twenty-four countries (Table 2, data from the NOURISHING database^(10)^). Sixteen countries had mandatory standards for TFA and twelve on salt. In comparison, voluntary reformulation standards were found in thirty-six countries. As well as salt and TFA, these voluntary policies also targeted sugar, saturated fat and (total) fat. More than half (sixteen) of the countries with mandatory policies also had voluntary policies. In some of these countries, the voluntary and mandatory policies target the same nutrient. For example, in Argentina, a voluntary agreement exists for salt content in dairy products, but mandatory salt targets exist for meat products, starchy products, soups and bouillons. In addition to nationwide policies listed in Table 3, some mandatory reformulation policies have been adopted at a regional level. For example, TFA restrictions, or standards for branded foods sold in schools, were implemented regionally in the USA before being implemented nationally.

**Table 2.** List of countries where mandatory and/or voluntary reformulation policies are implemented, data from the NOURISHING database as of December 2023 (World Cancer Research Fund International 2018)

Country	Mandatory reformulation policies	Voluntary reformulation policies (any focus)
Argentina	Salt, TFA	Yes (salt)
Australia	None	Yes (salt, saturated fat or sugar)
Austria	TFA	Yes (salt)
Belgium	Salt	Yes (salt)
Brazil	None	Yes (salt)
Bulgaria	Salt	Yes (salt, saturated fat or sugar)
Canada	TFA	Yes (salt)
Chile	TFA	Yes (salt)
Colombia	TFA	None
Costa Rica	None	Yes (salt)
Croatia	Salt	Yes (salt)
Czech Republic	None	Yes (salt)
Denmark	TFA	None
Ecuador	TFA	Yes (salt)
Estonia	None	Yes (salt, saturated fat or sugar)
Finland	None	Yes (sugar)
France	None	Yes (total fat, salt and sugar)
Germany	None	Yes (sugar, total fat and salt)
Greece	Salt	Yes (sugar, TFA and salt)
Hungary	Salt, TFA	Yes (salt)
Iceland	TFA	None
India	TFA	None
Iran	Salt, TFA	None
Ireland	None	Yes (salt)
Italy	None	Yes (salt, sugar, TFA and saturated fat)
Kuwait	None	Yes (salt)
Latvia	TFA	Yes (sugar, TFA and salt)
Lithuania	None	Yes (sugar, fat and salt)
Malaysia	None	Yes (salt)
Mexico	None	Yes (salt)
The Netherlands	Salt	Yes (salt and saturated fats)
New Zealand	None	Yes (salt and saturated fats)
Norway	None	Yes (salt, sugar and saturated fats)
Paraguay	Salt	None
Poland	None	Yes (sugar, total fat and salt)
Portugal	Salt	Yes (sugar, salt and TFA)
Singapore	TFA	None
South Africa	Salt, TFA	None
South Korea	None	Yes (salt)
Spain	Salt	Yes (total fat, salt, saturated fat, sugar and TFA
Switzerland	TFA	Yes (fat, salt and sugar)
UK	None	Yes (sugar, salt, saturated fat and TFA)
Uruguay	None	Yes (salt)
USA	TFA	Yes (salt)

TFA, *trans*-fatty acids.

**Table 3.** List of countries having implemented and evaluated the impact of a mandatory reformulation policy

Country	Foods targeted	Target of the policy	Implementation start	Evaluation
Denmark	All foods	TFA	2003	^(13,19)^
USA, New York States	Restaurant foods	TFA	2006	^(16,20,21)^
Austria	All foods	TFA	2009	^(22)^
The Netherlands	Bread	Salt	2009	^(23)^
NYC, USA	Branded foods in schools	Nutrient profile (several nutrients)	2010	^(24)^
USA, Massachusetts	Branded foods in schools	Nutrient profile (several nutrients)	2012	^(25,26)^
Argentina	All foods	TFA	2014	^(27)^
Argentina	Some food categories	Salt	2014	^(4)^
USA	Branded foods in schools	Nutrient profile (several nutrients)	2014	^(28)^
South Africa	All foods	Salt	2016	^(3–10,13,16,19–30)^

TFA, *trans*-fatty acid.

Only a small number of the mandatory reformulation policies implemented were evaluated (ten out of twenty-seven interventions, Table 3). Evaluations were mostly completed in the USA and in Europe, on policies focusing on salt or TFA. In comparison, the evaluation of voluntary reformulation policies was found for eighteen countries. Evaluation of voluntary reformulation policies focused mostly, as mandatory policies, on salt and TFA but also on several nutrients together, sugars or energy (Table 4).

**Table 4.** Summary of studies evaluating mandatory or voluntary reformulation

Country	Description of the intervention	Type of incentive	Study focus	Outcome*				Reference
				Food composition	Acceptability	Intakes	Morbidity/Mortality	
Argentina	From 2014, TFA limits 2 % of fats in oils and margarines and 5 % in other foods	Mandatory limit (standards)	TFA	+				^(27)^
	Mandatory salt limits, by category. Passed in December 2013, came into force in December 2014. Limit for three categories	Mandatory limit (standards)	Salt	+/−				^(4)^
Australia	Health Star Rating labelling: voluntary since 2014, supported by Australia and New Zealand governments	Information to consumers	Energy	+				^(31)^
	Food and Health Dialogue in 2009 (framework for governments, public health groups and industry to work together, including voluntary targets on salt)	Voluntary reformulation	Salt	–				^(32)^
				+				^(33)^
						–		^(34)^
	Energy labelling in restaurants	Information to consumers	Energy	–				^(35)^
	Reformulation of salt in one brand of bread (large-scale experiment)	Voluntary reformulation	Salt		+	–		^(36)^
Austria	Mandatory labelling of TFA contents	Information to consumers	TFA		+			^(37)^
	TFA restriction (< 2 % total fat) for processed foods since 2009	Mandatory limit (standards)	TFA				–	^(22)^
	Beverage checklist initiative monitoring and disclosing the sugar content of drinks	Information to consumers	Sugar	+				^(38)^
Brazil	Voluntary agreement to gradually reduce salt in foods	Voluntary reformulation	Salt	+				^(39)^
	Mandatory labelling of TFA contents	Information to consumers	TFA	+				^(27)^
				+				^(40)^
Canada	Mandatory labelling of TFA contents	Information to consumers	TFA	+				^(41)^
						+		^(42)^
						+		^(43)^
	Voluntary targets for salt reduction	Voluntary reformulation	Salt	+				^(44)^
Costa-Rica	Strategic alliance between government, academia and industries. Commitment from manufacturers to reformulate	Voluntary reformulation	TFA			+		^(45)^
							+	^(46)^
	Mandatory labelling of TFA contents	Information to consumers	TFA	+				^(27)^
Denmark	Ban on TFA from 2003 (passed on 1 January 2014)	Mandatory limit (standards)	TFA	+				^(19)^
							+	^(13)^
	Public–private partnership to increase whole grains in products	Voluntary reformulation	Whole grains	+		+		^(47)^
	Multicomponent initiative including voluntary reformulation	Voluntary reformulation	Salt			+		^(34)^
	Supermarket-led reformulation	Voluntary reformulation	Several nutrients	+	+			^(48)^
Finland	Multicomponent initiative including voluntary reformulation	Voluntary reformulation	Salt			+		^(34)^
France	Multicomponent initiative on salt since 2001 (public health campaign, reformulation and food procurement policies in some institutions)	Voluntary reformulation	Salt			+		^(34)^
	Public–private partnership defining reformulation targets for various nutrients	Voluntary reformulation	Salt		+/-			^(49)^
			Sugars		+/-			
			Several nutrients		+/-			
			Fibres		–			^(50)^
			Several nutrients		+			
			Salt		+			
			Sugars		–			
Ireland	Multicomponent initiative on salt since 2003 (public health campaign, reformulation and nutrition labelling)	Voluntary reformulation	Salt			+		^(34)^
Mexico	Mandatory labelling of TFA contents	Information to consumers	TFA	+				^(27)^
New Zealand	Health Star Rating (front-of-pack labelling using a nutrient profile model) since 2015	Information to consumers	Several nutrients		+			^(51)^
			Salt		+			
			Energy		+			
			Fibres		+			
The Netherlands	Choices front-of-pack labelling system: voluntary labelling since 2006	Information to consumers	Several nutrients	+				^(52)^
	Multicomponent intervention to reduce salt intakes, including targets for reformulation	Voluntary reformulation	Salt			–		^(34)^
	Mandatory limits for salt in bread (gradual reduction from 2009, latest amendment in 2013), several industry engagement to reduce salt in their foods	Voluntary reformulation (standards + voluntary)	Salt	+		–		^(23)^
Slovenia	Awareness campaigns on TFA	Information to consumers	TFA		+			^(53)^
Spain	Salt reduction in breads (voluntary reformulation by a manufacturer)	Voluntary reformulation	Salt		+			^(54)^
Switzerland	Multicomponent intervention, including voluntary reformulation targets	Voluntary reformulation	Salt			–		^(34)^
Turkey	Multicomponent initiative on salt since 2011 (public health campaign, reformulation and food procurement policies in some institutions)	Voluntary reformulation	Salt			+		^(34)^
UK	Salt reduction programme from 2004 (voluntary reformulation targets, public health campaigns and nutrition labelling)	Voluntary reformulation	Salt		+	+		^(55)^
						+		^(56)^
						+		^(57)^
							+	^(58)^
						+		^(34)^
						+		^(59)^
						+		^(60)^
	Sugar reduction programme from 2016 (voluntary reformulation targets, public health campaigns and nutrition labelling)	Voluntary reformulation	Several nutrients		+			^(61)^
			sugars		+			
	Voluntary reformulation of TFA	Voluntary reformulation	TFA			+		^(62)^
	Soft Drinks Industry Levy	Voluntary reformulation	Sugar	+				^(63)^
USA, California	Restriction on toy giveaways in restaurants	Information to consumers	Several nutrients	–				^(64)^
				–				^(65)^
USA, New York States	Review of the various policies implemented by NYC’s Dept of Education for school lunch and branded foods	Mandatory targets	Nutrient profile (several nutrients)					^(24)^
	TFA restriction in fast-food restaurants from 2006 in NYC	Mandatory limit (standards)	TFA	+	+			^(20)^
							+	^(16)^
							+	^(21)^
USA, Massachusetts	Nutrition standards for branded foods sold in schools from Massachusetts in 2012–2013 (similar to the nationwide standards to be implemented in 2014–2015)	Mandatory limit (standards)	Nutrient profile (several nutrients)	+/-				^(25) (26)^
USA	Mandatory labelling of TFA in packaged foods	Information to consumers	TFA			+		^(66)^
				+				^(67)^
	Energy labelling in restaurants	Information to consumers	Energy	+/-				^(68)^
				+/-				^(69)^
				+/-				^(70)^
				+/-				^(71)^
				+				^(72)^
				–				^(73)^
	National salt reduction initiative	Voluntary reformulation	Salt		+			^(74)^
					+			^(75)^
					+			
					+			
					+	+		^(76)^
					+	+		^(77,78)^
						–		
				–				^(79)^
				–				^(80)^
	Voluntary reformulation commitment from restaurants	Voluntary reformulation	Several nutrients	–				^(81)^
	Healthy, Hunger-Free Kids Act of 2010 (HHFKA) (standards for reimbursable school meals and standards for competitive foods)	Mandatory targets	Nutrient profile (several nutrients)	+				^(28)^○
	New dietary guidelines on whole grain intakes in 2005	Voluntary reformulation (raised awareness)	Whole grains		+			^(82)^
South Africa	Mandatory targets on salt content. Signed in 2013. First deadline in 2016 and second deadline in 2019	Mandatory limit (standards)	Salt	+				^(14)^
				+				^(85)^
				+				^(3)^
						+		^(30)^
						+/-		^(83)^

TFA, *trans*-fatty acid.

Blanks mean that the outcome was not evaluated.

* Changes in the outcomes were rated as positive (+), negative (-) or mixed (+/-). Positive changes were defined as a significant change in average nutrient density or a change in nutrient intake going in the direction of an improvement for public health (e.g. reduction for salt, TFA, energy or sugars, increase in fibres), or a reduction in disease risk or mortality.

### Design and implementation of mandatory reformulation policies

Based on the literature examined in this review, the different mandatory reformulation policies launched around the world appear to share some common features in terms of design and implementation. Namely, their launch followed a stepwise process, starting with the identification of the issue, the search for the best solution and a stakeholder consultation.

#### Identification of a diet-related public health issue

The first step in the design of a mandatory reformulation policy was often the identification of a public health issue with dietary causes. Three studies mentioned that the potential health impact of reformulation was an argument used to implement the policy. For example, the fact that the consumption of TFA brings no nutritional benefit but is linked to ‘considerable potential harm’, for example, an increased risk of coronary heart disease pushed many countries to set up a ban on TFA^(11,12)^. In Denmark, the ban was introduced in 2003 when research showed that the mean TFA intake was 1 g/d in 2001^(13)^ and that 1 % of the Danish population was at higher risk due to consumption of more than 5 g/d^(13)^. In South Africa, it was recognised that hypertension rates were increasing, and the current dietary guidelines were not sufficient to limit the intake of high-salt foods. The identification of this problem was a crucial step in the decision to implement mandatory standards for salt^(14)^.

#### Identification of solutions

Three studies described the implementation approach for the mandatory reformulation policy. Once the problem has been identified, different solutions that could help dealing with the problem (reformulation as an option among different solutions, e.g. marketing and advertising restrictions, labelling initiatives, public awareness, …) must be considered to ensure that the most effective policy is chosen. When an adverse health outcome can be linked to a specific nutrient, for example, a high intake of salt increasing the risk of hypertension, the main food sources of this nutrient to be targeted by the policy are identified. An illustration of this process is the implementation of the mandatory TFA restrictions in the USA. First, TFA were banned from restaurant foods in New York City from 2006. Fried foods were identified as a large contributor of TFA in the diet. As a lot of fried foods came from restaurants, and as restaurants provided a third of daily energy content in New York City, they were chosen to be the target of the policy^(15)^. It was concluded that restricting TFA in restaurants had the potential to have a meaningful impact on dietary intakes of the population^(15)^. Other cities and jurisdictions followed New York City’s example and implemented similar mandatory restriction on TFA^(16)^. The implementation of the restrictions was linked to a 6·2 % reduction in cardiovascular disease (CVD)^(16)^. It was only later, in 2015, that the US Food and Drug Administration recognised TFA as no longer ‘generally recognised as safe’ and hence banned TFA from all foods sold in the USA, including in the retail sector.

A total ban as used for TFA is not possible for other nutrients, for example, salt/Na as they cannot be removed totally from the diet. Na, for example, is generally consumed in excess but cannot be removed from the diet completely for health reasons, nor removed totally from food as it often plays a sensory and/or technical role. Na is the second most frequently targeted nutrient in mandatory reformulation policies. In Na reduction policies, standards or target levels for salt/Na content are set dependent on food categories and generally focus on the categories contributing the most to intakes. The targets or standards are set following careful consideration of where the balance lies between the feasibility of reformulation for that category and the potential for reformulation to reduce Na intakes. As bread has been identified as a main contributor to salt intakes across Europe, generally due to the amount consumed rather than high levels in products, some countries have implemented a mandatory target or standard for bread only^(17)^.

Two studies reported that a mandatory reformulation policy was implemented after the failure of other approaches to improve identified public health problems^(15,17,18)^. Before the implementation of the mandatory TFA restrictions in New York City restaurants, an education campaign was in place between 2005 and 2006 to help restaurants reduce their use of TFA^(15)^. However, this voluntary policy did not lead to a change in the percentage of restaurants using oil containing TFA^(15)^. Hence, the mandatory restriction was implemented to force restaurant owners to change their practices.

In Argentina, the mandatory policy was implemented based on the structure of a previous voluntary policy^(18)^. The mandatory salt standards were defined based on previously existing standards used in a voluntary public–private partnership. The voluntary standards devised through the partnership had the advantage of being achievable by the industrial partners who were involved in their design. However, a study that assessed the mandatory standards concluded that they were not challenging enough^(18)^ This conclusion was drawn because there was a high level of compliance in some categories shortly after implementation, due to the same standards having been used in the previous voluntary reformulation programme, suggesting that the mandatory targets did not incentivise much additional reformulation. Therefore in 2018, Argentina decided to strengthen the standards and reduce the targets by 5 % for meat and farinaceous products to further challenge food producers. In 2019, the standards for bouillons and soups were lowered to keep the reformulation efforts ongoing^(4)^.

#### Engagement with stakeholders

Two studies reported the role of engaging stakeholders in the development, design and implementation of the mandatory reformulation policy. This is a recognised step in constructing voluntary reformulation policies to maximise engagement by businesses across all sectors^(4–10,13,14,16,19–84)^.

In South Africa, the process included a consultative phase with stakeholders, including industry, although industry did not feel this process was consultative enough and felt the outcome was already decided before the consultation^(14)^. It is likely that the more stringent standards set in South Africa, compared with Argentina, resulted from a process where industry had less ability to exert influence. Further evaluations are needed to understand the impact of the different level of engagement of industry stakeholders on the reformulation efforts and compliance, as well as on dietary behaviours and intakes, and health outcomes.

Stakeholders were also involved in the design of a policy, as described in the making of the New York City school food standards for branded foods sold in schools^(24)^. Students and school staff were involved to gauge and fully understand the feasibility and acceptability of the initiative^(24)^.

### Effectiveness of mandatory policies on nutrient composition

Showing that a policy improves the nutrient composition of foods is one of the most important steps to demonstrate its effectiveness. Ten studies evaluated the impact of eight mandatory reformulation policies on the composition of foods (Table 4). Overall, these studies showed that the mandatory policies were effective in improving the composition of targeted foods. However, some nuances must be discussed.

First, all studies showed an improvement in the nutrient profile of targeted food products sold after the implementation of a mandatory reformulation policy. Bans on TFA in the USA and in Argentina were effective in removing TFA from the food supply^(11,12,14,15,27,28,30–85)^. Argentina and South Africa implemented mandatory standards on salt for a large part of their food supply in 2014 and 2016, respectively. In both countries, the announcement and implementation of the targets were linked to an increase in the proportion of products complying with the standards^(3,4)^. In Argentina, the number of products compliant with the mandatory targets increased from 85 % to 90 %, even though the same targets already existed before as voluntary targets^(4–86)^.

These studies showed that mandatory reformulation policies may be effective in improving the levels of the targeted nutrient in targeted foods. However, these studies often limit their evaluation to the nutrient targeted by the policy, while they do not evaluate changes in the whole nutrient profile of foods^(11,12,15,17,18,85–87)^.

Standards for the nutrient profile of foods sold in schools were also effective in leading to improvements in those foods^(26–28)^.

### Effects of mandatory policies on consumer-related outcomes

Evaluating the impact of a nutrition policy on consumer-related outcomes takes into consideration consumer preferences and the effect that the policy could have on dietary intakes and health. Six studies were found that evaluated the impact of mandatory reformulation policies on consumer-related outcomes (Table 4).

Four studies evaluated changes in consumer intake or purchases of the nutrient targeted by the reformulation policy^(16,20–23)^. The quantities of TFA in fast food’s purchased decreased after the implementation of the TFA ban in New York City^(20)^. Restaurants with high TFA usage changed their practices and as a result, customers’ purchase of TFA decreased. Two studies showed a decrease in population salt intakes in South Africa following the introduction of salt targets, one of which showed a significant decrease (–0·2 g salt/d in a 4-years’ time frame)^(83,84)^. However, there was no change in salt intake in the Netherlands between 2011 and 2016, when both mandatory and voluntary salt standards were put in place^(23)^. Despite some reduction in the salt content of foods, it was not enough to improve the Dutch population intake of salt. Reasons include the reformulation of only a few number of categories and relatively low baseline levels of Na intakes in the Netherlands^(23)^.

Investigation of the impact of the Argentinian salt targets on dietary intake (measured using food records or urinary Na measurement) was not carried out.

Four studies investigated the impact of TFA bans on the health status of populations in Denmark and in New York State. These studies, using robust methods including synthetic control cohorts or difference-in-differences designs, showed that the implementation of a ban on TFA was associated with a reduction in CVD mortality by 4·3 % to 6·2 % after 3 or more years^(13,16,20)^. However, a similar study on the effect of the Austrian ban on TFA showed no improvement in CVD mortality^(22)^. The study provided an explanation for the lack of change in CVD mortality: contrary to what happened in the other countries that formed part of the control group, smoking prevalence increased in Austria^(22)^. Smoking is a known risk factor for CVD, and it is very likely that an increase in smoking prevalence would counterbalance health benefits brought by a ban on TFA.

Despite several studies on the implementation of mandatory standards for branded foods in school, no study has evaluated the impact of these policies on children’s intake, which remains a significant gap in the literature.

### Consequences of mandatory reformulation policies on businesses

Only four studies covering three US policies, and the policy in South Africa, considered the impact on businesses.

The time between the policy announcement and the implementation of the policy varied, depending on the strictness of the standards and the time needed by businesses to reformulate their products to be compliant with the new standards. In Argentina, manufacturers were given 2 years to comply with the mandatory salt standards, which were already being used by some manufacturers via previous a voluntary agreement^(4)^. In South Africa, two sets of standards were introduced to be achieved within 3 and 5 years, respectively. The 5-year deadline was delayed by a year to give more time to manufacturers to reformulate their products^(14)^. In New York City, restaurants had 6 months to change the oils and spreads they used, and 18 months for other items, to comply with the ban on TFA^(20)^. The short phase-out period for oils was justified by an education campaign that had been in place 1 year before the ban that supported restaurants to use a TFA-free oil.

Reformulating foods can sometimes be costly for manufacturers as it can require funds for research and development, and adjustments in production processes^(14,85)^. Although reformulating recipes is part of business as usual for manufacturers, some may need additional support and resource to reformulate their products to adhere to the nutrient targets of a new regulation. Specifically, small businesses may not have in-house budget and technical capacities to engage with the reformulation process^(27)^.

The implementation of standards on branded foods sold in schools was linked to an initial loss of revenue from these products for schools^(88)^. This policy was implemented together with nutrition standards for canteen meals, which led to an increase in the demand for those meals. In the second year of the policy, the total revenues rebounded to levels before the policy was implemented because of increased sales of the meals. As well as giving schools time to adapt to the new guidelines, the transition phase also gave manufacturers time to reformulate if they wanted to continue to sell their branded products in schools. In Massachusetts, 17 % of branded foods sold in schools were reformulated during a 2-year period after enactment of the law in 2012^(26)^. In comparison, 8·3 % of branded foods were repackaged in smaller packs in the same time frame to make them compliant with the per-portion standard.

### Changes in the composition of foods in the absence of any policy

One study found that the nutritional quality of foods of a country improved in the absence of any implemented reformulation policy. The study by Monge-Rojas in four Latin American cities found that reformulation to remove TFA from fast-food products occurred before any legislation was introduced^(27)^. The authors suggested that the reformulation occurred due to recommendations to reduce TFA in the diet, or because transnational companies were reformulating their products globally, in response to voluntary or mandatory reformulation programmes implemented elsewhere. Countries that implemented mandatory reformulation policies have done so because it was the only solution to effectively drive a change in the amount of the targeted nutrient in the desired direction.

### Limitations to 100 % compliance of reformulation policies

In contrast to voluntary reformulation programmes, mandatory reformulation policies are, in theory, less dependent on manufacturers’ will to improve their products. This should result in an improvement in the nutrient composition of the foods targeted by the policy, and a high proportion of compliant products should be observed. However, mandatory reformulation does not always produce as large an effect as expected, and eight studies reported limitations to compliance for two policies.

Studies on the mandatory standards in Argentina showed that compliance was 85 % at the introduction of the law^(86)^ and at 90 % 4 years later^(4)^. First, this shows that even in the presence of a mandatory policy, not all food will be reformulated to comply. Salt content in Argentinian foods remained quite high, despite compliance with the standards. Researchers suggested that the standards were not challenging enough, and they recommended that the targets should be readjusted to incentivise further reformulation^(4)^. Since the initial implementation of the targets, some were strengthened to incentivise further reformulation.

In South Africa, compliance was also not at 100 % 3 years after the implementation of the mandatory salt standards. Research showed that compliance was at 72 % for the 110 products sampled^(3)^. The lower overall compliance rate in South Africa compared with Argentina can be explained by the fact that the South African targets were more ambitious, thus more difficult to achieve, than the Argentinian ones. Compliance varied by food categories, with high compliance for uncured (but not cured) meat, breakfast cereals and noodles^(3–10,13,16,19–30)^. In crisps and salt and vinegar crisps, set as a separate category from other flavours of crisps because of their high salt content, less than 30 % of products complied. Denmark, the first country to ban TFA from its food supply from 2004, also had non-compliant foods^(19)^. Looking at the dynamics of TFA removal from food products, authors of the study suggested that it may have been more difficult for small- and medium-sized food companies to reformulate than for larger food companies^(19)^.

In both countries, the implementation of the standards was enforced using fines for non-complying companies^(89,90)^. However, the fines were not mentioned in later evaluations. In South Africa, fines were supposed to be enforced by local authorities or municipalities, who may not have been used to having this kind of oversight^(19)^.

### Effectiveness of mandatory reformulation policies compared to other policies

Mandatory reformulation policies can be difficult to accept in more liberal economies. Some perceive it to create disparities between smaller and larger food companies. On the one hand, larger food manufacturers often have more knowledge and financial capacity to reformulate their products. On the other hand, big companies feel they carry a disproportionate burden in reformulation, as it is easier to monitor compliance of big companies compared with smaller ones.

**Fig. 2.**
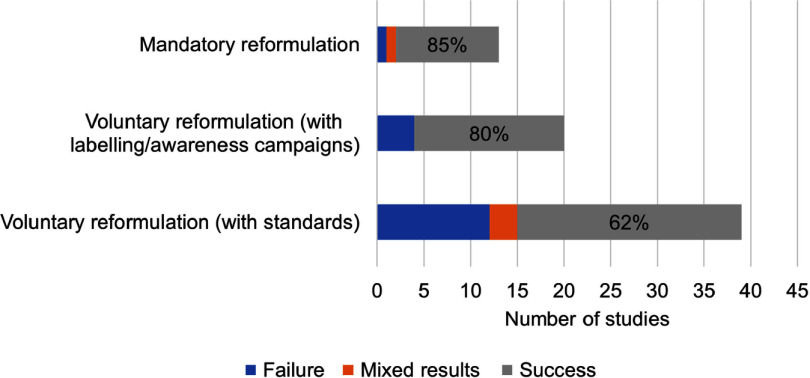
Numbers of studies available and effectiveness^1^ of different incentives promoting reformulation on improving the food environment, consumer purchases, intakes or health. ^1^The percentages indicate the proportion of studies included in this review showing successful results. Changes in the outcomes were rated as success, failure or mixed results. Success was defined as a significant change in average nutrient density or a change in nutrient intake going in the direction of an improvement for public health (e.g. reduction for sodium, TFA, energy or sugar and increase in fibre), or a reduction in disease risk or mortality. Failure was defined as an absence of change, or a change in the unexpected direction. Mixed results mean both success and failure (e.g. for different categories). TFA, *trans*-fatty acids.

Policies that highlighted the nutrient content of a product to consumers, such as labelling, as an incentive for manufacturers to reformulate foods had a relatively high rate of success in driving reformulation compared with voluntary reformulation standards alone (80 % effectiveness when information if given to consumers *v*. 62 % effectiveness with voluntary standards, Fig. 2). These policies gave information to consumers through voluntary or mandatory labelling of the nutrition content of products. Such disclosure of information created an incentive for manufacturers to (voluntarily) reformulate their products. The disclosure of foods’ nutrient content was also used by civil society groups and was associated with the reformulation of products^(38–53)^.

## Discussion and Conclusions

This review compares the effectiveness of mandatory *v*. voluntary food reformulation policies targeting different nutrients and implemented in different countries. Mandatory reformulation policies appear to be more effective in driving reformulation than voluntary policies. As a result, such policies are likely to be more effective in improving the composition of foods (relating to the nutrient targeted) and dietary intakes of the relevant nutrient. More research should be done to evaluate the impact on intakes and health outcomes of all reformulation policies (voluntary and mandatory).

The factors behind the effectiveness of mandatory reformulation policies could help improve voluntary reformulation policies. Strict standards for voluntary reformulation suffer from lower compliance but may hold more promise for improving the levels of the nutrient of concern. Strict monitoring can be implemented to reassure manufacturers of a level-playing field. If well implemented, and with sufficient monitoring, a mandatory reformulation policy should incentivise more reformulation than a voluntary policy. If technical advice is provided to manufacturers who may not have the in-house capacity to revise their recipes (e.g. small businesses), this may help to reduce barriers for all manufacturers and enable greater compliance with the targets.

### Strengths and limitations

The main limitation in this review is that the different policies examined were difficult to compare. More robust comparisons were possible when the same evaluation metric was used across countries, for example, the reduction in the salt content of foods compared between South Africa and Argentina. Nonetheless, it should be noted that the comparison of two policies implemented in two different countries can be biased by the way the policies were implemented, structured, enforced and their effect measured.

In addition, the evaluation of reformulation policies depends on the methods used to monitor the average nutrition composition of food products. Monitoring a change in the composition of foods raises some challenges, for example, food labels not being up to date, data being transcribed incorrectly and not corrected later^(91)^. Furthermore, products sold by smaller businesses can be more difficult to monitor, especially if they are only sold locally^(11,12,14,15,17,18,85,86)^. Studies that assessed compliance to the law sampled foods from bigger cities, and it is unlikely that a study could possibly sample all foods sold by local producers. The lack of data was identified as a limitation to scale up salt reduction programmes in these countries^(92)^.

A further limitation of this review is that the risk of bias assessment was not performed for all included studies. Furthermore, it showed that most studies did not evaluate the effect of reformulation independently from other policies that were implemented around the same time that may also have prompted reformulation. In addition, this study highlighted that the impact of many mandatory reformulation policies had not been assessed (or the assessment was not published).

This review suggests that using features that make mandatory policies effective (e.g. strict standards and strict monitoring) to design voluntary reformulation policies could make them stronger and more impactful. Nonetheless, the consequences of a voluntary reformulation policy with strict standards and monitoring cannot be derived from this review. This review can help develop an optimal policy informed by lessons learned from other countries but should be adapted to the local context.

### Comparison with previous reviews

Comparing evidence from this review on mandatory and voluntary reformulation policies shows that mandatory reformulation had a higher success in improving food composition than other policies (Fig. 2). Other systematic reviews found the same conclusion^(93,94)^. Other policies included voluntary reformulation policies like reformulation targets or labelling laws. None of the above-mentioned systematic reviews found that voluntary reformulation policies would be more effective than mandatory policies in improving the nutrient content of foods.

## Supporting information

Gressier et al. supplementary material Gressier et al. supplementary material

## Supplementary material

For supplementary material/s referred to in this article, please visit https://doi.org/10.1017/S0007114524001326
