# Supplementary material for: The effectiveness of mandatory v. voluntary food reformulation policies: a rapid review
Source: Br J Nutr. 2025 Mar 28;133(6):737–50. doi: 10.1017/S0007114524001326 (PMC12169955; doi:10.1017/S0007114524001326)
Supplement: Gressier et al. supplementary material [file S0007114524001326sup001.docx]

# On-line Supplementary Material

Evidence review on mandatory reformulation policies: what is their effect and how do they compare to voluntary reformulation policies?

Authors: Mathilde Gressier, Gary Frost, Zoe Hill, Danying Li, Jack Olney, Elisa Pineda, Victoria Targett, Michelle Young, Franco Sassi

# Supplementary tables

Supplementary table 1 : Search strategy run on MEDLINE

| 1 | sodium, dietary/ or Sodium Chloride/ or sodium chloride, dietary/ or exp ENERGY INTAKE/ or dietary fats/ or Fats, Unsaturated/ or Fatty Acids, Unsaturated/ or Dietary Sugars/ or Dietary fiber/ or exp Nutritive value/ or whole grains/ or dietary carbohydrates/ or dietary proteins/ |
| --- | --- |
| 2 | (salt or sodium or sugar* or energy or calori* or saturated fat* or "trans fat*" or trans?fat* or fibre* or fiber* or whole?grain* or wholegrain* or "whole grain*" or carbohydrate* or protein* or fatty acid* or nutrient*).ab. |
| 3 | 1 or 2 |
| 4 | reformulat*.ab. |
| 5 | 3 and 4 |
| 6 | ((salt or sodium or sugar* or saturated fat* or "trans fat*" or trans?fat or TFA) adj5 (target* or limit* or restrict* or regulat* or reduc*)).ab. |
| 7 | ((fibre* or fiber* or whole?grain* or wholegrain* or whole grain*) adj5 (improv* or increas* or promot* or favo?r*)).ab. |
| 8 | ((energy or calori*) adj5 (reduc* or limit* or target*)).ab. |
| 9 | (((improve* or better or enhance* or health*) adj5 (composition* or profile*)) and (nutrition or food or nutrient)).ab. |
| 10 | 5 or 6 or 7 or 8 or 9 |
| 11 | (sold or sales or intake* or purchase* or consumption or diet* or overweight or diabetes or bmi or cholesterol or "coronary heart disease" or cardiovascular or "dietary habit*" or "heart disease risk" or "consumer behaviour" or "consumer behavior" or "blood pressure" or hyperglyc?emia or "glucose tolerance" or "insulin resistance" or hypertension or hyperlipidemia or dyslipidemia).ab. |
| 12 | (grocery or groceries or store or stores or supermarket or supermarkets or retailer or retailers or market or markets or food industry or food dispensers or vending or point-of-purchase or point-of-selection or package* or packages or front-of-pack).ab. |
| 13 | ((regulat* or polic* or legislation* pledge* or ban or bans or standard or standards or strategy or strategies or intervention* or restriction*) and food*).ab. |
| 14 | 12 or 13 |
| 15 | exp animals/ not humans.sh. |
| 16 | (restaurant* or fast-food* or "fast food*" or fastfood* or takeaway* or take-away* or "take-away*").ab. |
| 17 | 14 or 16 |
| 18 | 10 and 11 and 17 |
| 19 | 18 not 15 |

Supplementary table 2: Risk of bias of studies included in this review also included in the published review by Gressier et al., 2021

|  | Risk of bias score^1^ | Reference number^2^ |
| --- | --- | --- |
| Grabovac, 2018 | **6** | (17) |
| Ratnayake, 2014 | **3** | (38) |
| Friesen, 2006 | **2** | (39) |
| Monge-Rojas, 2013 | **2** | (41) |
| Colón-Ramos, 2006 | **6** | (42) |
| Restrepo, 2016 | **7** | (13) |
| Jensen, 2017 | **4** | (44) |
| Oqali, 2016 | **3** | (45) |
| Spiteri, 2018 | **6** | (46) |
| Ni Mhurchu, 2017 | **4** | (47) |
| Temme, 2017 | **4** | (18) |
| Zupanič, 2018 | **2** | (49) |
| Quilez, 2016 | **3** | (50) |
| Eyles, 2013 | **6** | (51) |
| Griffith, 2017 | **6** | (52) |
| He, 2014 | **5** | (53) |
| He, 2014 | **5** | (54) |
| Millett, 2012 | **4** | (55) |
| Hutchinson, 2018 | **6** | (58) |
| Angell, 2012 | **6** | (14) |
| Brandt, 2012 | **7** | (15) |
| Restrepo, 2016 | **7** | (16) |
| Vesper, 2017 | **6** | (62) |
| Ahuja, 2015 | **3** | (70) |
| Clapp, 2018 | **4** | (71) |
| Curtis, 2016 | **3** | (72) |
| McLaren, 2016 | **5** | (30) |
| Mancino, 2008 | **4** | (78) |
| McMahon, 2017 | **5** | (32) |
| Henninger, 1996 | **2** | (33) |

^1^ The risk of bias score was obtained using the Newcastle Ottawa Scale.

^2^ Reference numbers refer to the reference list of the main manuscript
